# Frequency and Correlates of Anxiety Symptoms in Serbian Primary Care

**DOI:** 10.1192/j.eurpsy.2025.1497

**Published:** 2025-08-26

**Authors:** M. Spasić Stojaković, M. Latas

**Affiliations:** 1 Psychiatry, Kliniken im Theodor-Wenzel-Werk, Berlin, Germany; 2 Faculty of Medicine, Belgrade University, Belgrade, Serbia; 3 Psychiatry Depratment, Faculty of Medicine, Belgrade University; 4 Clinic of Psychiatry, University Clinical Centre of Serbia, Belgrade, Serbia

## Abstract

**Introduction:**

Globally, anxiety disorders are ranked as the sixth largest contributor to non-fatal health loss (WHO, 2017). Data on anxiety in primary care patients in low- and middle-income countries are sparse.

**Objectives:**

Determining prevalence and demographic correlates of symptoms of anxiety in primary care across Serbia.

**Methods:**

Cross-sectional study was conducted in 100 primary care facilities from whole Serbia. Sample consisted of 10-12 consecutive patients interviewed by each of the 270 partaking general practitioners, total of 2041. The participants answered sociodemographic questionnaire, and if they were previously diagnosed with F40 or F41. GAD-7 questionnaire was used for the assessment of symptoms of anxiety (cut-off score ≥ 10). Multiple logistic regression was applied.

**Results:**

Positive screening for anxiety was found in 22.7% of participants. It was significantly associated with gender (male OR: 0.769, 95% CI: 0.602-0.982), marital status (divorced OR: 1.901, 95% CI: 1.346-2.686; single OR: 1.573, 95% CI: 1.042-2.375), education (high school OR: 0.578 95%, CI: 0.410-0.814; university OR: 0.489, 95% CI: 0.323-0.740), employment (unemployed OR: 1.903, 95% CI: 1.355-2.672; retired OR: 1.797, 95% CI: 1.209-2.672; homemaker OR: 3.018, 95% CI: 1.842-4.945) and region of residence (Beograd OR: 0.595, 95% CI:0.430-0.824; Central Serbia OR: 0.371, 95% CI:0.231-0.597) Percentage of participants who have screened positively for anxiety without having previously established anxiety (F40, F41) diagnosis was 61.4%.

**Conclusions:**

Anxiety is relatively prevalent among patients in primary care in Serbia and it is associated with gender, marital status, education, employment, and region of residence, with homemakers, divorced and the ones with only primary school being the most likely to have positive screening for anxiety. Even though positive screening for anxiety as measured here does not necessarily imply diagnosis of anxiety disorder, it nevertheless, means that the person experiences distress, and may need clinical attention - substantial percentage of patients with positive screening for anxiety is unrecognized and untreated.

**Disclosure of Interest:**

None Declared

